# Population Variability Generated during Rescue Process and Passaging of Recombinant Mumps Viruses

**DOI:** 10.3390/v13122550

**Published:** 2021-12-20

**Authors:** Anamarija Slović, Tanja Košutić-Gulija, Dubravko Forčić, Maja Šantak, Maja Jagušić, Mirna Jurković, Dorotea Pali, Jelena Ivančić-Jelečki

**Affiliations:** 1 Centre for Research and Knowledge Transfer in Biotechnology, University of Zagreb, 10000 Zagreb, Croatia; aslovic@unizg.hr (A.S.); tkgulija@unizg.hr (T.K.-G.); dforcic@unizg.hr (D.F.); mjagusic@unizg.hr (M.J.); mjurkovic@unizg.hr (M.J.); dpali@unizg.hr (D.P.); 2 Ruđer Bošković Institute, 10000 Zagreb, Croatia; maja.santak@irb.hr

**Keywords:** virus rescue, mumps virus, virus variability

## Abstract

Recombinant mumps viruses (MuVs) based on established vaccine strains represent attractive vector candidates as they have known track records for high efficacy and the viral genome does not integrate in the host cells. We developed a rescue system based on the consensus sequence of the L-Zagreb vaccine and generated seven different recombinant MuVs by (a) insertion of one or two additional transcription units (ATUs), (b) lengthening of a noncoding region to the extent that the longest noncoding region in MuV genome is created, or (c) replacement of original L-Zagreb sequences with sequences rich in CG and AT dinucleotides. All viruses were successfully rescued and faithfully matched sequences of input plasmids. In primary rescued stocks, low percentages of heterogeneous positions were found (maximum 0.12%) and substitutions were predominantly obtained in minor variants, with maximally four substitutions seen in consensus. ATUs did not accumulate more mutations than the natural MuV genes. Six substitutions characteristic for recombinant viruses generated in our system were defined, as they repetitively occurred during rescue processes. In subsequent passaging of primary rescue stocks in Vero cells, different inconsistencies within quasispecies structures were observed. In order to assure that unwanted mutations did not emerge and accumulate, sub-consensus variability should be closely monitored. As we show for Pro408Leu mutation in L gene and a stop codon in one of ATUs, positively selected variants can rise to frequencies over 85% in only few passages.

## 1. Introduction

Reverse genetics technology enables recovery of infectious, replication-competent RNA virions from plasmids with cloned complementary DNA (cDNA) and engineering of RNA viruses with specific genetic properties. Such de novo production (rescue) of viruses from cDNA template encoding complete viral genome, with or without additional transcription units (ATUs), is a valuable tool in basic research of virus biology as well as in vaccine development and design of therapeutic biologicals.

Among non-segmented negative-sense RNA viruses, viruses produced using reverse genetics have been mostly based on the measles virus [1,2] or vesicular stomatitis virus [3], although biotechnological platforms that enable viral rescue have also been developed for many others including *Mumps orthorubulavirus* [4].

*Mumps orthorubulavirus* (named “the mumps virus” until it was changed by the International Committee on Taxonomy of Viruses in 2015; MuV) is a member of the *Orthorubulavirus* genus within the *Paramyxoviridae* family. The virions are enveloped, pleomorphic particles of variable size. MuV genome is 15,384 nucleotides long and packed in helical nucleocapsid. It contains seven genes: the nucleocapsid (N), phospho- (P), matrix (M), fusion (F), small hydrophobic (SH), hemagglutinin-neuraminidase (HN) and large (L) protein gene [5,6,7]. Besides phosphoprotein, P gene encodes two more proteins, V and I. In nature, MuVs follow “the rule of six” principle [8] and maintain proper N-phasing context [9].

MuV is a human pathogen that causes a systemic, vaccine-preventable illness spread by respiratory route. The infection is asymptomatic in approximately one third of cases [10]; the disease is generally self-limiting and characterized by parotitis and mild nonspecific symptoms, although the virus has the capacity to invade several visceral organs and central nervous system. Since the late 1960s, mumps has been prevented by vaccination. All vaccines against mumps are based on live attenuated strains. A number of MuV vaccine strains have been developed, but only a few are still used nowadays [11]; one of them is L-Zagreb [12]. Obtaining the optimal attenuation level and vaccine efficacy has proven to be a challenging task for MuV vaccines [11,13]. Due to the neuropathogenic properties inherent to MuV, a number of mumps vaccines showed insufficient safety profiles and were withdrawn from the market. Despite continuing research efforts, the genetic determinants of MuV neurovirulence are still unknown.

The ability to manipulate MuV genome by reverse genetics systems has been used as a tool (a) for investigation of MuV biology [14,15,16,17,18,19,20,21], (b) for optimization of a mumps vaccine [22], and (c) to investigate possibilities of using MuV as a vector in recombinant vaccines or in gene therapy [4,23,24,25,26].

RNA viruses produced by rescue methodology are often referred to as viruses derived from infectious clone [26,27], implying that high genetic consistency of viral populations can be achieved using this technology. Still, all populations of RNA viruses, including recombinant, always exist as swarms of variants (collectively termed quasispecies) that are genetically linked through mutations and together contribute to the population characteristics [28].

We have established a rescue system based on the consensus sequence of the L-Zagreb vaccine. Recombinant attenuated MuVs based on established vaccine strains represent attractive vector candidates, as they have track records of high efficacy and the viral genome does not integrate into the host cells due to the fact of their cytoplasmic replication. In this research, our aims were (a) to characterize the level of viral population diversity that arises during the rescue process; (b) to identify variants inherent to recombinant viruses generated in our rescue system; (c) to analyze further diversification of viral populations that occurs after the rescue process is finished and passaging is carried out under usual in vitro conditions. We also analyzed whether ATUs accumulated more mutations than natural MuV genes as they are not necessary for viral life cycle.

## 2. Materials and Methods

### 2.1. Cells

Vero cells were obtained from the European Collection of Authenticated Cell Culture. BSR T7/5 cells were a kind gift from Klaus Conzelmann (Gene Center, Ludwig Maximilian University of Munich). BSR T7/5 is a BHK-derived cell line stably expressing T7 RNA polymerase under control of the cytomegalovirus promoter and the neomycin resistance gene [29].

Vero cells were maintained in Eagle’s Minimum Essential Medium (MEM); BSR T7/5 in Dulbecco′s Modified-MEM (D-MEM). Growing media were supplemented with 10% fetal bovine serum (FBS; PanBiotech) and penicillin (100 U/mL)—streptomycin (100 µg/mL) (Capricorn). During cultivation of BSR T7/5, 0.01 g/mL of G 418 disulfate salt (Merck) was added in every second passage. Cultures were kept at 37 °C in a humidified atmosphere of 5% CO_2_.

### 2.2. Plasmids

The rescue process was based on concomitant transfection of BSR T7/5 cells with four plasmids. Three are supporting plasmids that express the MuV nucleoprotein, phosphoprotein and large protein, the fourth expresses complete MuV antigenomic sequence. All MuV sequences were based on the consensus sequence of L-Zagreb vaccine strain (NCBI GenBank acc. no. AY685920).

Supporting plasmids in rescue experiments (pSG5-N, pSG5-P and pSG5-L) were generated by cloning the corresponding genes’ 5′ noncoding regions and the entire coding regions (MuV cDNA nucleotide positions 56–1795, 1909–3152 and 8430–15223 for the N, P and L gene, respectively) into pSG5 (Agilent Technologies, Santa Clara, CA, USA). Plasmid containing the MuV P gene was modified by insertion of two guanosines in the MuV editing site (MuV cDNA nucleotide position 2439–2444).

**Figure 1:**
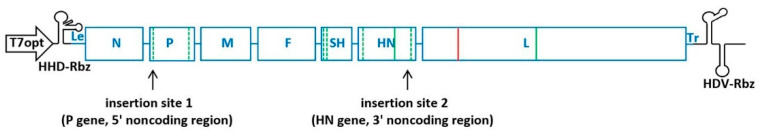
Partial scheme of pMRV2, showing positions of optimized T7 promoter (T7opt), hammerhead ribozyme (HHD-Rbz), complete mumps antigenomic sequence (in blue; consisting of leader sequence (Le), 7 genes (the nucleocapsid (N), phospho- (P), matrix (M), fusion (F), small hydrophobic (SH), hemagglutinin-neuraminidase (HN) and large (L) protein gene), intergenic regions and trailer sequence (Tr); not drawn strictly to scale) and hepatitis delta virus ribozyme (HDV-Rbz). Green and red lines indicate positions of differences to L-Zagreb vaccine consensus sequence. Dashed green lines indicate differences in noncoding regions (T1923C, A3175G, T6240A, A6241C and T8391G; the first letter indicates nucleotide identity in L-Zagreb vaccine consensus sequence) full green lines indicate synonymous substitutions (C8008T and C11176T), full red line indicates the nonsynonymous substitution (T9660C). The two insertion sites that were used in construction of plasmids with additional genomic segments are indicated.

Our basic plasmid that contained the complete MuV antigenome was pMRV2 (its full sequence is available from NCBI GenBank acc. no. MZ964863). The backbone segment of pMRV2 was derived from 286_p107(-)Luc, a kind gift from Branka Horvat and Denis Gerlier from Centre International de Recherche en Infectiologie, Lyon. Complete antigenomic sequence of the L-Zagreb vaccine consensus was inserted by sequential cloning and subcloning of cDNA fragments using endogenous or primer introduced restriction enzyme sites. It was positioned between (a) optimized T7 promoter and hammerhead ribozyme sequence [4] and (b) hepatitis delta virus ribozyme sequence [4] positioned directly upstream and downstream of the antigenome, respectively (Figure 1). The sequence of pMRV2 differs in nine nucleotides compared to L-Zagreb consensus. Seven differences were deliberately introduced during the cloning process in order to create additional restriction sites, while the two differences in the L gene coding region occurred coincidentally: one is T9660C, leading to the amino acid change Leu408Pro; the other is synonymous substitution C11176T.

The other six plasmids that contained complete viral antigenomes (shown schematically in Figure S1) were derived from pMRV2 by (a) insertion of one or two ATUs at the insertion site 1 (in the 5′ noncoding region (NCR) of the P gene (Figure 1)); (b) insertion of an ATU at insertion site 1 and an 84-nucleotides long random sequence at insertion site 2 (located in the HN gene 3′ NCR); (c) replacement of L-Zagreb consensus sequence with alternative synonymous sequences.

In plasmids that possessed ATUs added using site 1, complete inserts were constructed in a way that the coding sequences were flanked by P gene 5′ NCR and N gene 3′ NCR upstream and downstream of the coding region, respectively. In plasmids with alternative synonymous sequences, partial P or complete SH coding sequences in MuV antigenome were changed by introducing numerous synonymous changes in order to maximize the number of CG and AT dinucleotides. This potentially facilitated the recognition of viral RNAs by innate immunity receptors [30].

In construction of all plasmids with complete viral sequences, the rule of six as well as proper N-phasing context were strictly adhered to. For plasmid with additional ATU(s) between the N and P genes, ATU(s) were in the P gene N-phase.

Plasmids used for this study were the following:

(1) pMRV2;
(2) pMRV3—possessed the enhanced green fluorescent protein (EGFP) gene inserted into insertion site 1;
(3) pF–RSV-MRV2—contained the ATU consisting of sequence coding for *Human orthopneumovirus* (RSV, from previous name human respiratory syncytial virus) fusion protein ectodomain fused to transmembrane and cytoplasmic domains of MuV F protein (F–RSV); this ATU is added using insertion site 1;
(4) pE1E2TMD–HCV–MRV2 contains two ATUs, consisting of sequences coding for *Hepacivirus C* (HCV, from previous name Hepatitis C virus) E1 or E2 ectodomain, each fused to transmembrane and cytoplasmic domains of MuV F protein; these ATUs are added using insertion site 1;
(5) p*mi*scr-MRV3 was derived from pMRV3, by insertion of an 84-nucleotides long random, noncoding sequence at insertion site 2; this created the longest noncoding region in the MuV genome;
(6) pVdeopti-MRV2 possesses an alternative synonymous sequence of MuV genomic region 2001–2416, that codes for the protein segment common to P, V and I proteins;
(7) pSHdeopti-MRV2 possesses alternative synonymous sequence of the SH gene coding region.

Sequences are available at NCBI GenBank, under acc. nos. MZ929423, MZ929424, MZ964861-MZ964864 and OK001340.

Plasmids were propagated in *E. coli* DH5α. Overnight cultures of colonies were grown in LB medium (Carl Roth) supplemented with 100 µg/mL of ampicillin (Merck) at 37 °C and 120 rpm. Plasmids were isolated with ZR Plasmid Miniprep-Classic kit (Zymo Research).

### 2.3. Generation of Recombinant Viruses

The BSR T7/5 cells were seeded at concentration 0.6 × 10^6^ cells/well in 6-well plates and were grown for 24 h at 37 °C with 3 mL of growth medium with 10% FBS per well. Four hours before transfection, the medium was replaced with a fresh aliquot. Before transfection, cells were washed 2× with OPTI-MEM (ThermoFisher Scientific, Waltham, MA, USA), and 0.8 mL of OPTI-MEM was added to each well.

Transfection mixtures contained: 1 µg of pSG5-N, 0.5 µg of pSG5-P, 1 µg of pSG5-L, 5–10 µg of plasmid with complete viral antigenomic sequence, 5 µL of Plus reagent, 9 µL of Lipofectamine LTX (both from ThermoFisher Scientific) and 300 µL of OPTI-MEM. The mixtures were added drop by drop onto cells.

Transfected cells were incubated for 18 h at 37 °C. The following day supernatants were removed, cells were washed 2× with OPTI-MEM and 3 mL of fresh growth medium (D-MEM with 10% FBS) were added. Cells were incubated at 37 °C and monitored for the appearance of cytopathogenic effect (CPE) for the next 5–7 days. Afterwards, BSR T7/5 cells from a single well were transferred to 25 cm^2^ flasks and 10 mL of D-MEM with 3% FBS was added. Cells were incubated at 35 °C for the next 3 days. Cells were divided in a ratio 1:3 or 1:6 every 3 or 4 days until extensive CPE was detected (duringeach rescue process, these sub-cultivations were performed 1–3×). At that point, supernatant was collected and propagated further in Vero cells for one or two passages, until again extensive CPE was observed.

Viruses MRV3 and F–RSV-MRV2 have undergone somewhat different passage histories. After two passages in Vero cells, due to the extremely hardly noticeable CPE, MRV3 was grown for one additional passage in BSR T7/5, and then for one more in Vero cells.

During rescue process of F–RSV-MRV2, virus from BSR T7/5 supernatant was plaque purified as described in Kosutic-Gulija et al. [31]. Single plaques were picked and propagated in Vero cells. Supernatant from Vero cells with most distinctive CPE was chosen for further processing.

Primary rescue stocks were prepared by an additional passage in Vero cells. Virus titers were determined (as described in Forcic et al. [32]) in pre-stock seeds and while in suspension, Vero cells were infected at MOI 0.0001. Cells were seeded and incubated at 35 °C in a medium with 5% FBS until extensive CPE was observed (7–10 days). Supernatants were collected, centrifuged for 10 min at 2000× *g*, aliquoted and stored at −60 °C. Obtained primary rescue stocks possessed the following titers (in logCCID_50_/mL): MRV2 7.47, MRV3 8.15, F–RSV-MRV2 6.79, E1E2TMD-HCV-MRV2 6.36, *mi*scr-MRV3 6.16, Vdeopti-MRV2 5.72 and SHdeopti-MRV2 7.83.

### 2.4. Virus Passaging

Ten blind serial passages of MRV2, MRV3 and F–RSV-MRV2 were conducted in the Vero cell line. Specifically, 1 mL of MRV2 was mixed with 4 × 10^6^ cells, cells were seeded in 25 cm^2^ flask and incubated at 35 °C for 5–7 days in growth medium with 5% FBS. When extensive CPE was observed, supernatant was collected, and 1 mL was used as the inoculum for the next passage. MRV3 and F–RSV-MRV2 were serially passaged with 1 µL or 100 µL of inoculum (passaging series are named A or B, respectively). Inoculum was mixed with 1 × 10^6^ cells, cells were seeded in 25 cm^2^ flask and incubated at 35 °C for 7 days in growth medium with 2% FBS. Supernatants were collected, 1 or 100 µL was used as the inoculum for the next passage.

### 2.5. Viral RNA Isolation, Reverse Transcription and PCR Amplification

Viral RNA was extracted from 400 µL of cell culture supernatant using Quick-RNA Viral Kit (Zymo Research, Irvine, CA, USA) following manufacturer’s recommendations. Isolated RNA was reversely transcribed and cDNA amplified following one of the two protocols: reverse transcription was done with Superscript III (ThermoFisher Scientific) and cDNA amplification with Phusion polymerase (New England Biolabs, Ipswich, MA, USA), or RNA was reversely transcribed with M-MLV (Invitrogen, Waltham, MA, USA) and cDNA was amplified using Velocity polymerase (Meridian Bioscience, Cincinnati, OH, USA). In both protocols, reaction mixtures and PCR regimes were as recommended by enzyme manufacturers. For some samples, higher DNA yield and less nonspecific amplification was obtained with the first protocol, while the others were amplified better following the second. As there were no data indicating that the two reverse transcriptases were different in transcription accuracy and the accuracies of the two polymerases were comparable [33], the two procedures were used interchangeably. The complete viral genomes were amplified in 4 or 5 overlapping fragments. The enzymes and primers used in preparation of each individual sample are shown in Tables S1 and S2.

PCR amplicons were separated on 1% agarose gels, excised, purified using Nucleospin Gel and PCR Clean-up Kit (Macherey–Nagel, Allentown, PA, United States) and quantified on Quantus fluorometer (Promega, Madison, WI, USA) with QuantiFluor^®^ ONE dsDNA System (Promega).

### 2.6. Next Generation Sequencing (NGS)

As starting samples for preparation of libraries, we used isolated plasmids or pools of overlapping PCR amplicons (mixed in equimolar amounts) covering complete viral genomes.

Libraries were prepared using Illumina DNA Prep kit (Illumina, San Diego, CA, USA) following the manufacturer’s protocol. The quality of the libraries was checked on a 2100 Bioanalyzer (Agilent) using the High Sensitivity DNA Kit (Agilent). Libraries were pooled and sequenced on an Illumina MiniSeq (Illumina), MiniSeq Mid Output Kit (2 × 150 paired-end reads, Illumina).

The quality of the raw reads was assessed with a FastQC v0.11.8 and subjected to adapter removal, trimming of bases below Q-score 30, and removal of reads shorter than 50 bp using BBDuk within the BBTools package. Paired-end reads were aligned to reference plasmid sequence using Bowtie2 v2.4.2 [34] with a high gap opening and extension penalty. Samtools v1.12 [35] was used for further processing of alignments, including removing reads that had more than two mismatches to reference sequence. Data regarding the number of obtained reads before and after filtering as well as mean coverage of alignment is shown in Table S3. Geneious Prime^®^ 2019.2.3 software was used for majority consensus calling and V-Phaser 2 [36] was used for inferring diversity within viral populations. Heterogeneous genomic positions were defined with our in-house Python script which removes variants in primer regions. Additionally, the script filters out those variants that are not present in at least five forward and five reverse reads.

NGS data are available from the NCBI SRA database, BioProject ID PRJNA769261.

### 2.7. Compilation of Genomic Sequences of Viruses Belonging to Genus Orthorubulavirus

We retrieved from NCBI GenBank database all publicly available sequences of the L gene of viruses belonging to genus *Orthorubulavirus* (8 species; data were retrieved on 15 June 2021). Following number of sequences were retrieved: 59 for *Human orthorubulavirus 2*, 28 *for Human orthorubulavirus 4*, 9 for *Mammalian orthorubulavirus 5*, 2 for *Mammalian orthorubulavirus 6*, 2 for *Mapuera orthorubulavirus*, 310 for MuV, 7 for *Porcine orthorubulavirus* and 2 for *Simian orthorubulavirus*.

### 2.8. L Protein Structure Prediction

L protein structure was predicted using the automated protein structure homology-modeling server SWISS-MODEL [37], available at http://swissmodel.expasy.org and visualized using Geneious Prime^®^ 2019.2.3.

## 3. Results

### 3.1. Homogeneity of Input Plasmids

In order to ascertain that the variability we observed in viral populations did not stem from the variability of input plasmids (i.e., plasmids with antigenomic viral sequences used in rescue experiments), we analyzed the plasmids’ homogeneity by NGS. The plasmids were not PCR amplified prior to NGS, other than five cycles that were part of the NGS library preparation.

Plasmid samples possessed none or only few heterogeneous positions (data not shown). The highest percentage of a variant in all input plasmid samples was 0.44%, detected in plasmid pE1E2TMD–HCV–MRV2 for a variant possessing C at genomic position 2751. That was the only heterogeneity seen in input plasmids that was also obtained in the resulting rescued virus: in primary rescue stock of E1E2TMD–HCV–MRV2, the minor variant possessing C was present in 4.06% of reads, possibly being present due to the variability in the input plasmid.

All other minority variants were obtained only in the input plasmids and in lower percentages. They were not further investigated.

### 3.2. Threshold for Detection of Heterogeneous Genomic Positions in Viral Samples

Unlike plasmid samples, viral cDNAs were PCR amplified prior to NGS. In order to distinguish low-frequency variants from variants caused by errors introduced during PCR amplification and sequencing, we amplified the region of pMRV2 corresponding to MuV antigenome and used these amplicons for NGS library preparation.

Amplicons were prepared in triplicate. Heterogeneity was found at 0, 10 and 24 positions. The highest mismatch frequency (the sum of percentages of all variants detected at a single genomic position) was 0.99. Based on this result, we set the variability threshold of our method for virus samples at 1%.

### 3.3. The Variability of Viral Populations in Primary Rescue Stocks

The first goal of this research was to characterize the level of population diversity that arises during the rescue processes. We analyzed which mutations occur and to what extent do the obtained viral populations differ from input plasmid sequences.

**Figure 2:**
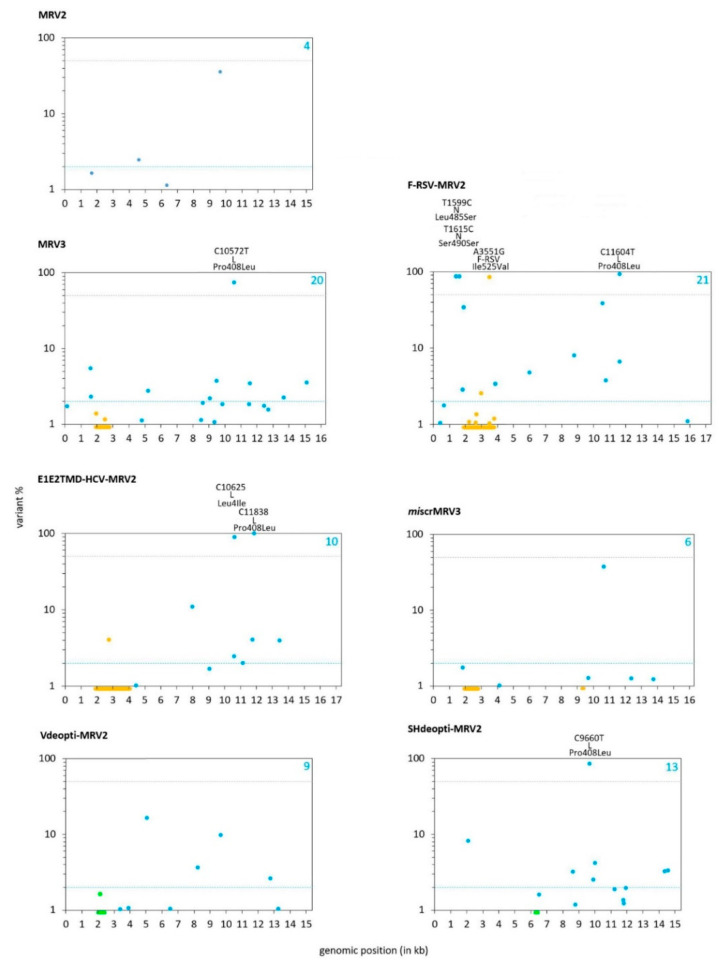
Heterogenous genomic positions and variants’ frequencies in viral primary rescue stocks; the number of heterogenous positions is shown in each graph in the upper right corner. Blue and grey dashed lines emphasize 2% and 50%, respectively. Changes in viral consensus sequences (relative to input plasmid sequences) are specified above each graph (the first nucleotide/amino acid is the identity in plasmid); nucleotide change, protein and amino acid change are shown. The regions added to the mumps genome and the regions with alternative synonymous sequences are indicated below the *x*-axis in orange and green, respectively. The dots that indicate heterogeneities in those regions are also shown in orange or green.

All seven recombinant MuVs were successfully rescued and grew to high titers. Viral genomes in primary rescue stocks were quite homogenous; variability above 1% was obtained at 4–21 genomic positions (Figure 2; Table 1, Tables S4 and S5). Only nucleotide substitutions (not indels) were detected. In total, 48% of heterogeneous sites possessed variant frequencies in the range 1–2%.

Compared to sequence of input plasmids, the population consensus was changed in four viruses, at one position in MRV3 and SHdeopti-MRV2, two in E1E2TMD-HCV-MRV2, and four in F–RSV-MRV2 (Figure 2, Table 1, Tables S4 and S5; variants present in frequencies higher than 50% were part of population consensus). In all these four different recombinant viruses, the same nucleotide substitution leading to a Pro→Leu change of amino acid 408 in the L protein occurred in consensus (further presented in Section 3.6).

Four of the recombinant MuVs contained genomic inserts. Few heterogeneous positions were observed in inserts: none in *mi*scr-MRV3, one in E1E2TMD–HCV–MRV2 (stemming from heterogeneity of input plasmid), two in MRV3 and seven in F–RSV-MRV2 (Figure 2, Table 1 and Table S5). Unlike others, nonsynonymous substitution A3551G in F–RSV-MRV2 occurred at a high percentage (79.02%), leading to amino acid change Ile525Val. F–RSV-MRV2 was the only virus that was plaque purified during the rescue process. Four plaques with different morphologies were picked and individually propagated; the one that was chosen for primary rescue stock establishment was the one with most distinctive CPE. Viral populations from the other three plaques were subjected to different NSG analysis, not fully comparable to the one presented in this paper. The position 3551 was not detected as heterogeneous in any of them, it was identical as in input plasmid (data not shown). Sequences of wild-type RSV fusion glycoproteins possessing both Ile and Val at amino acid position 525 can be retrieved from NCBI database.

Compared to segment’s length, slightly higher number of heterogeneous positions was observed in F–RSV-MRV2 ATU; their frequency in this insert was 3.6 × 10^−3^ or 0.36%. The maximum value calculated for any of the other MuV genes in our set of primary rescue stocks was 0.32%, obtained in N and SH genes. The frequency of heterogeneous positions in E1E2TMD–HCV–MRV2 ATU was 0.05, in MRV3 ATU it was 0.22.

Taken together, these data show that generated MuV populations faithfully match sequences of input plasmids. Low percentages of heterogeneous positions were obtained and differences from plasmid sequences were predominantly seen in minority variants. ATU regions did not differ in that aspect from natural MuV genes. Still, few high frequency mutations (reflected in populations’ consensus) did occur during virus generation, in the course of only few rounds of genome transcription (by T7 polymerase) and replication (by viral RdRp) necessary to complete the rescue process.

### 3.4. Heterogeneous Genomic Positions Common to Different Recombinant MuVs

The second goal of this research was to determine whether the same variants arose in different recombinant MuVs. In viruses with inserts, genomic positions analogous to positions in non-lengthened genomes were located further downstream. In this analysis we disregarded the inserts and adjusted the ordinal numbers of nucleotides to corresponding genomic positions in viruses possessing natural MuV genome length of 15,384 nucleotides.

**Figure 3:**
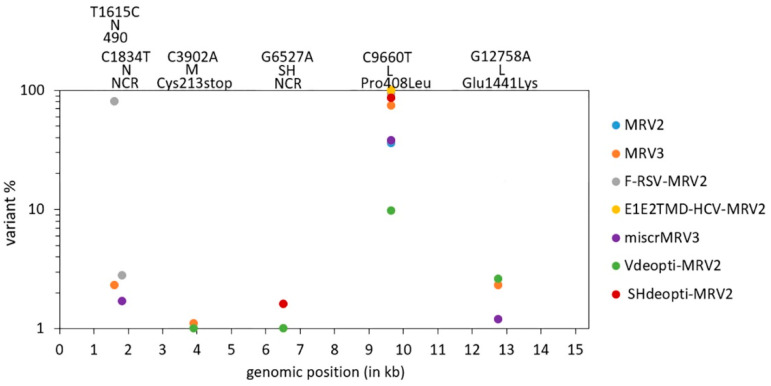
Positions and percentages of six substitutions common to at least two primary viral rescue stocks. The changes indicate the difference from input plasmid sequence (the first nucleotide/amino acid is the identity in plasmid). The nucleotide change, gene and amino acid change are indicated, except for the synonymous substitution T1615C, where only the number of corresponding amino acid is shown, and for substitutions that occurred in noncoding region (NCR). For MRV3, F–RSV-MRV2, E1E2TMD-HCV-MRV2 and *mi*scrMRV3 only the regions of mumps virus backbone (not the insert(s)) were included in the analysis, their genomic positions are adjusted to corresponding positions in MRV2.

Six heterogeneous positions were common for at least two rescued viruses (Figure 3). In all viruses, heterogeneity was observed at genomic position 9660 in the L gene, where high percentages of mutated variants (coding for Leu as amino acid 408) were present. At other five identified positions, variants were detected in frequencies below 3%. Regardless of differences in their abundance, as these heterogeneities repetitively occurred in different viruses, we regard them as characteristic for viruses generated in this experimental setup.

In the following experiments, ten additional passages of three primary rescue stock viruses (MRV2, MRV3 and F–RSV-MRV2) were performed. Two of the variants defined as inherent to our rescue system were detected during passaging in viruses that did not originally possess them, strengthening the conclusion that these mutations do not occur randomly. The substitution C3902A was seen during passaging of F–RSV-MRV2 and substitution G6527A during passaging of MRV2 and MRV3.

### 3.5. Population Variability Generated under Usual In Vitro Passaging Conditions

During the rescue process, MuV genome synthesis is carried out under specific conditions: transcription from plasmid is performed by T7 RNA polymerase; early rounds of MuV replication by RdRp occur in an environment different from normal virus infection in a host cell [25]. By obtaining high titer primary rescue stocks, we considered the rescue processes to be completed. Further passaging was performed in order to investigate to what extent MuV quasispecies will change now, as the replication occurs under usual in vitro conditions. As a cellular substrate, we chose Vero cells that are conventionally used for MuV propagation or cell-based assays [38].

We performed ten consecutive passages of three primary rescue stock viruses (MRV2, MRV3 and F–RSV-MRV2) and analyzed the variability of populations after passages no. 5 and 10. These three viruses were chosen because preparation of their primary rescue stocks differed: MRV2 was passaged minimally in BSR T7/5 and Vero; MRV3 was obtained after three cell culture changes (rescue was done in BSR T7/5, virus was propagated in Vero, then, again, in BSR T7/5 and back in Vero); F–RSV-MRV2 underwent a plaque purification step prior to stock preparation. Passaging of MRV3 and F–RSV-MRV2 was done twice, in two independent series.

**Figure 4:**
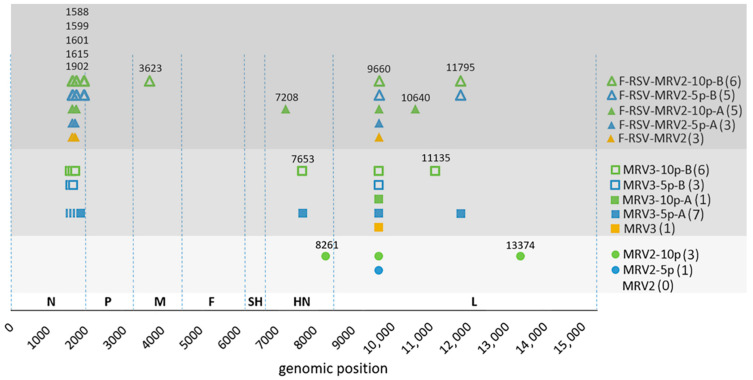
Position of substitutions in viral consensus sequences relative to input plasmids. The number of substitutions is indicated in parenthesis next to viral sample name; no differences in consensus were found in MRV2. Only the regions of mumps virus backbone (not the inserts) were included in this analysis; the genomic positions for MRV3 and F–RSV–MRV2 were adjusted to corresponding positions in MRV2. Gene identities are shown above the horizontal line that represents the MuV antigenomic sequence in corresponding input plasmids.

The lowest number of heterogeneous genomic positions was found in MRV2 populations, while the diversities of MRV3 and F–RSV-MRV2 were comparable (Table 2). Again, all detected mutations were substitutions. The substitutions that were detected in consensus sequences of viral populations in primary rescue stocks were retained in consensus sequences of subsequent passages. That was not the case with mutations that occurred during passaging: compared to variability of populations in primary rescue stocks, the number of heterogeneous genomic positions increased by passage 5 and similar number was maintained in passage 10 (Table 2), but inconsistencies in the variants’ positions were observed (Table 2, indicated in columns “Number. of New Heterogeneous Sites”; Table S6). For both viruses that were passaged in two independent series, the two resulting populations in the same passage (in case of both passages no. 5 and no. 10) differed at several genomic positions, even at consensus level (max. 5, Table 3, Figure 4; note: in Figure 4 positions of substitutions in MRV3 and F–RSV-MRV2 are adjusted to correspond to equivalent positions in MRV2, ATU regions are omitted).

Similar to the populations’ variabilities that occurred during the rescue process, a substantial number of substitutions (39.7% in total) were detected in less than 2% of NGS reads. The only low-frequency mutation that was observed in both passages 5 and 10 within the same passaging series and in two different viruses was C3902A in M gene, leading to a premature stop codon after amino acid 212 (it is positioned at nucleotide 3902 in MuV genome if inserts are excluded from numbering; in Table S6, the corresponding positions are 4814 in MRV3 and 5846 in F–RSV-MRV2). Variant 3902A was found in primary rescue stock of MRV3 but not in F–RSV-MRV2. During passaging, we detected it in one passaging series of MRV3 and in both of F–RSV-MRV2. The maximum percentage of this variant during passaging was 1.71.

High-frequency variants (the ones that became part of populations’ consensus) were obtained in N, HN, L and F–RSV genes, with the exception of one synonymous substitution in M gene (Table 3, Figure 4). Four consensus substitutions were observed in total in N gene and all were clustered within a short genomic segment near the 3′ gene end (region 1588–1902). Two of them were common to two different viruses (Table 3, substitutions indicated with superscripts 3 and 4).

We did not obtain higher number of heterogeneous positions in ATU regions, neither in F–RSV gene (which codes for a protein that could be included in viral particles) in F–RSV-MRV2, nor in EGFP (which codes for cytosolic protein) in MRV3. In F–RSV-MRV2 passaging line A, mutation G2066T leading to stop codon after amino acid 29 was observed in passage 5 in F–RSV gene (Table S6). This mutation was positively selected, its frequency was 41.99% in passage 5 and 87.86% in passage 10. The 2066T variant was not detected in primary rescue stock of F–RSV-MRV2 or in viral populations from passaging B series. This was the only substitution leading to stop codon detected in ATUs.

To summarize: (a) fluctuations within the quasispecies structure were observed during passaging and that was even reflected in the population consensus but only to a minor degree (at maximally six genomic positions); all consensus substitutions that occurred during rescue process were preserved during passaging; (b) heterogeneous genomic positions were detected across the entire genome length, high-frequency variants were observed in the N, M, HN, L and F–RSV genes.

### 3.6. Substitution C9660T in the L Gene

All input plasmids contained a cytidine at position 9660 (a position in the MuV genome if inserts are excluded from the numbering). This difference from the L-Zagreb vaccine consensus was unintentionally introduced during the cloning process of the basic plasmid pMRV2. The “reversion” to thymidine (a nucleotide present in L-Zagreb vaccine consensus), was observed in all primary rescue viral stocks as well as in all five passaging series, leading to Pro→Leu change of amino acid 408 in L protein (Figure 3 and Figure 4).

We created L protein models using SWISS MODEL server, one possessing Leu as amino acid 408, the other Pro (Figure S2). Models were based on the structure of parainfluenza virus 5 L–P complex, template 6v85.1.A [39]. L protein residues 5–2232 (98% of protein length) have been modeled with 0.66 ± 0.05 average model confidence (QMEANDisCo). Local confidence for residue 408 was 0.84 for Leu and 0.78 for Pro. According to the models, amino acid 408 was placed in the middle of an α-helix located close to RdRp domain (its position was mapped according to UniProtKB entry P30929).

Leu at amino acid position 408 was conserved in all 310 MuV entries publicly available in the NCBI GenBank database that covers this genomic region. In fact, when we inspected the identity of the corresponding amino acid in all available sequences of viruses belonging to genus *Orthorubulavirus* (besides MuV, there are seven other viral species in this genus), in all sequences, the amino acid corresponding to amino acid 408 in MuV L protein was Leu.

In primary rescue stocks, the frequency of variants possessing this substitution at position 9660 was in a range 9.8–100% (Figure 3, Table S4). During passaging, 9660T variant was also positively selected; its frequency had arisen in all viral populations, reaching minimally 98.65% in passage 10 (Table 3, substitutions indicated with superscript 1).

## 4. Discussion

In comparison to genomic properties of natural MuVs, different modifications were introduced during the construction of our recombinant viruses: insertion of one or two ATUs, encoding for cytoplasmic EGFP protein or different transmembrane recombinant proteins; lengthening of a noncoding region to the extent that the longest noncoding region in MuV genome was created; replacement of the original L-Zagreb genes’ consensus sequences with sequences rich in CG and AT dinucleotides. The fact that all viruses were successfully rescued and obtained in high titers indicates the plasticity of MuV genome and suggests its suitability as a platform for various biotechnological purposes. In our recombinant viruses with ATUs, all ATUs were placed between the N and P genes. Ammayappan et al. [26] have shown that in recombinant MuV ATUs between M and F genes or HN and L genes can also be expressed.

In construction of all our recombinant viruses, two paramyxoviral genomic rules were strictly adhered to: the rule of six and N-phasing. In the article by Ikegame et al. [18], only the rule of six was strictly followed. They presented results that show that the replication of MuVs possessing genomes with false N-phasing was not as successful and such viruses were quickly and overwhelmingly outgrown.

All mutations we detected during rescue processes and passaging were substitutions. For generation of final NGS data sets, on which we based the conclusions regarding mutations, we used settings that filtered a number of indels (high gap opening and extension penalty was used; reads that have more than two mismatches to reference sequence were removed). We repeated the data analysis without these filters. We did observe indels, but only of a single nucleotide. Such indels have been previously detected during genetic manipulations with viruses which follow the rule of six [40,41,42,43], including MuV [44], but the majority of genome lengthenings in paramyxoviruses that occurred and persisted in nature are characterized by insertions of multiple nucleotides at a single insertion site [45,46,47,48]. Moreover, in our alignments all indels were in homopolymeric stretches, which are known to be more prone to sequencing errors, especially regarding indels [49]. Therefore, we chose to adhere to more strict analysis parameters.

Another aspect of MuV populations should be born in mind when variability is analyzed, particularly in L gene: the observed variability could have partially originated due to the presence of 5′ copy-back type of defective interfering genomes (5′ cb DIGs). With the exception of E1E2TMD-HCV-MRV2, all viruses were amplified in a manner that the fragment covering 5′ genomic end was at least 3800 nucleotides long, therefore allowing amplifications of only longer 5′ cb DIGs. In our previous research regarding mumps 5′ cb DIGs that included three different mumps strains (Urabe, Jeryl Lynn 5 and 9218/Zg98), the longest mumps 5′ cb DIGs detected were 1344 nucleotides long [50]. Bosma et al. [51] analyzed 5′ cb DIGs in populations of recombinant mumps viruses based on 88–1961 strain. The longest mumps 5′ cb DIGs described by Bosma et al. [51] were 2878 nucleotides long, which is, again, shorter than our amplicons.

During the rescue processes, we obtained high genetic homogeneity of viral populations. Heterogeneities were detected at small number of genomic positions and in total, in approx. half of instances they occurred in frequencies below 2%. This is in line with the data observed in the field: generally, MuV is considered genetically rather stable, similar to measles virus [52], especially when compared to some other negative strand RNA viruses, like influenza or *Human orthopneumovirus*. We did not identify a genomic region that would be more prone to substitutions and we did not observe higher variability of inserted regions during the rescue process.

We were particularly interested in identifying common variants, which could be regarded as inherent to our rescue system. Given that our recombinant viruses have genomes of 15384–17562 nucleotides and that the maximum frequency of heterogeneous positions was 0.12% per genome, it seemed doubtful that the same variants would independently arise in different viruses by chance. Six substitutions were defined as repetitively occurring (i.e., characteristic for recombinant viruses generated in our rescue system): T1615C, C1834T, C3902A, G6527A, C9660T and G12758A. Substitution C9660T was likely necessary for RdRp functionality; the significance of others remains unknown. It is possible that their presence would ensure virus adaptation to different cellular environments, as it was shown for polio virus minority variant [53] necessary for neuroinvasion.

Substitution C3902A leads to a premature stop codon in M gene. It was found in two primary rescue stocks and it appeared during passaging of another one. It was always detected at low frequencies (the maximum was 1.71%). Long-term transmission of a minor variant with premature stop codon in envelope glycoprotein has been observed in nature, in dengue virus type 1 populations, possibly offering selective advantage for coinfecting viruses by ensuring the presence of viruses differentially adapted to various cell types, decoying host immune responses, or allowing the production of extra capsid and membrane proteins [54]. For MuV, in in vitro experiments, the existence and continuing replication of viral variants that rely on other variants in the pool to complement their genomic defects has been previously shown [18] but the biological significance of variants with shortened proteins has not been determined. Premature stop codons in M gene of measles virus are characteristic for strains that cause subacute sclerosing panencephalitis, a fatal progressive neurological disorder sometimes caused by wild-type measles strains [55]. MuV is also a neuroinvasive and a neurovirulent pathogen. Unlike measles, some vaccine MuV strains also possess these characteristics including L-Zagreb [56]. The genomic markers of MuV neuroinvasiveness and neurovirulence are unknown, and neither is known whether shortened M protein is somehow involved.

During passaging, substitutions T1615C and C1834T (identified as inherent to our system), as well as three others in the N gene were observed in high percentages. They were all positioned within a short genomic segment of 314 nucleotides (region 1588–1902), only two out of five being nonsynonymous. Plaque-purified recombinant MuVs with clusters of mutations in N or P gene were described before in a study by Bamford et al. [25]. They generated recombinant MuVs based on the consensus sequence of a wild type strain. In a population with clusters of mutations in N gene, the heterogeneities were grouped in genomic regions 607–860 and 1225–1558, there were 32 of them in total, but only one was nonsynonymous. Heterogeneous sites we detected were in a nearby downstream region. Ikegame et al. [18] also observed that recombinant MuVs preferentially permit mutations (which in their study were insertions) in N and P genes, especially in their noncoding regions. Whether changes in 5′ terminal region of N gene can have influence on the nature or quantity of defective interfering particles as has been shown for a point mutation in Sendai virus [57]; or influence mRNA transcription gradient as in measles virus [58] has yet not been shown for any MuV strain.

The strongest positively selected substitution in our rescue setup was the one in L gene, C9660T, leading to Pro→Leu change of amino acid 408 in L protein. This amino acid is located within the first of six blocks of high conservation defined for paramyxoviral L proteins [59]. Within genus *Orthorubulavirus*, only Leu has been detected at the amino acid position corresponding to amino acid 408 in MuV L protein. Paramyxoviruses belonging to other genera do not share this characteristic. A cysteine at position 9660 was undeliberately introduced instead of thymidine during cloning of pMRV2. Original L-Zagreb vaccine populations do not possess 9660C variant. In the past, we performed NGS of three L-Zagreb samples: master seed, working seed and a qualified viral pool produced at the Institute of Immunology Inc. We did not observe any variability at position 9660 (data not published). Due to the lack of crystallographic structure of MuV L protein, its structural predictions can only be made based on comparison with other related negative strand RNA viruses. According to MuV L protein modeling based on parainfluenza virus 5 L-P complex [39], amino acid 408 is located in the middle of an α-helix located closely to RdRp catalytic domain. Usually if there are prolines in helices, they introduce a bend and majority of them are situated in the first turn of helices or at positions where polypeptide chains change direction [60]. Whether Pro408 spatially negatively influences RdRp activity or specifically Leu408 is required for optimal activity of L protein is now being investigated. Nevertheless, the finding that the reversion C9660T was present in high percentages already in all primary rescue stocks demonstrates that selection pressures can strongly operate during the rescue process.

Upon generation of recombinant viruses, we monitored to what extent will the obtained populations further diversify during passaging in Vero cells. MRV2, MRV3 and F–RSV-MRV2 were chosen because during preparation of their primary rescue stocks all schemes most commonly met in master seed production of viral vaccines were included (minimal passaging (MRV2); multiple changes of cell substrate (MRV3); plaque purification (F–RSV-MRV2)).

As explained in results, Vero cells were chosen because they are conventionally used for MuV propagation or cell-based assays [38]. Vero cells lack interferon signaling [61] and antiviral reactions in them are significantly lessened. Whether this facilitates persistence and detection of biologically relevant variants or only the ones that are Vero-specific is unknown. Bamford et al. [25] and Ikegame et al. [18] that obtained results comparable to ours also used Vero as a cellular substrate. Still, it must be emphasized that a variety of factors, including innate immunity reactions, influence the variability of viral populations and the heterogeneities observed during passaging in Vero cells cannot be considered as general quasispecies characteristics of MuV strains.

Majority of heterogeneous positions detected during passaging were different both between the two passaging series of the same virus and between passages 5 and 10 within the same series. The resulting populations in the same passage differed even at consensus level at several genomic positions (maximum five). This argues against the idea that recombinant viruses might be produced without having to rely on seed stocks. Whether different substitutions/heterogeneities detected in populations during passaging are reflected in populations’ phenotype (e.g., in growth kinetics or specific infectivity) has not been investigated.

Some substitutions detected in passage five were not observed in passage 10. These may represent neutral changes and whether they become preserved is determined by chance. Another possibility is that they have accumulated due to genetic drift and eventually have been negatively selected, but this selection happened over a course of a few passages.

During passaging higher diversity was obtained in MRV3 and F–RSV-MRV2 than in MRV2. Besides the fact that preparations of their primary rescue stocks differed (possibly leading to differences in adaptation level to Vero cells), the reason for this might be that because MRV3 and F–RSV-MRV2 have an additional ATU, their basal variability level is different.

One of our goals was to determine whether ATUs will accumulate more mutations than the natural virus genes as they are not necessary for viral life cycle. This did not occur. Similar was previously observed in recombinant measles viruses where transgenes are stably expressed, although they are nonessential and, in some cases, even deleterious for virus propagation and are expected to be more rapidly mutated than the measles virus genes [62]. Still, in F–RSV gene, in one passaging line, a stop codon occurred after the first 29 amino acids. This variant was strongly positively selected during passaging in Vero cells. Similar has happened during clinical evaluation of MEDI-534, an RSV vaccine candidate based on chimeric bovine/human parainfluenza virus type 3 and expressing RSV F protein [63]. Only 2.5% of the virus in the clinical trial material used for human administration did not express RSV F but approx. half of the nasal wash specimens from vaccine recipients contained virus with mutations expected to reduce or ablate expression of the RSV F insert [63]. Although our choice of MuV as a vector was governed by the field data showing that MuV is genetically stable, in this experimental setup in vitro passaging of recombinant MuVs quickly introduced versatile sub-consensus variability that should be closely monitored in order to assure that unwanted mutations do not accumulate.

## Figures and Tables

**Table 1:** Number and distribution of heterogeneous sites in viral primary rescue stocks. Types of detected substitutions are also indicated.

	MRV2	MRV3	F–RSV-MRV2	E1E2TMD-HCV-MRV2	*mi*scr MRV3	Vdeopti-MRV2	SHdeopti-MRV2
Genome Length	15,384	16,296	17,328	17,562	16,380	15,384	15,384
Insert Length	n/a	912	1944	2178	996 ^a^	n/a	n/a
Total Number of Heterogeneous Sites (%)	4 (0.03)	20 (0.12)	21 (0.12)	10 (0.06)	6 (0.04)	9 (0.06)	13 (0.08)
in Coding Regions	4	17	17	8	4	8	12
Synonymous	0	5	6	2	0	0	0
Nonsynonymous, Missense	4	11	10	5	4	7	12
Nonsynonymous, Nonsense	0	1	1	1	0	1	0
in Noncoding Regions	0	3	4	2	2	1	1
Number of Heterogeneous Sites in Insert(s)	n/a	2	7	1	0	n/a	n/a
in Coding Regions		1	6	0			
Synonymous		0	0	0			
Nonsynonymous, Missense		1	6	0			
Nonsynonymous, Nonsense		0	0	0			
in Noncoding Regions		1	1	1			
Number of Changes in Consensus Sequence ^b^	0	1	4	2	0	0	1

^a^ Combined length of two inserts. ^b^ Relative to input plasmid sequence. Not applicable, n/a.

**Table 2:** Number of heterogeneous genomic positions in complete viral genomes (light grey) and solely in additional transcription unit (ATU) regions (dark grey) observed during passaging. For comparison, equivalent data for viral primary rescue stocks are also shown.

	In Complete Genome			In ATU		
Virus Sample	Number of Heterogeneous Sites (%)	Number of New ^a^ Heterogeneous Sites	Number of Changes ^b^ in Consensus	Number of Heterogeneous Sites	Number of New ^a^ Heterogeneous Sites	Number of Changes ^b^ in Consensus
MRV2	4 (0.03)	n/a	0	n/a	n/a	n/a
MRV2-5p	13 (0.08)	11	1	n/a	n/a	n/a
MRV2-10p	10 (0.07)	4	3	n/a	n/a	n/a
MRV3	20 (0.12)	n/a	1	2	n/a	0
MRV3-5p-A	30 (0.18)	27	7	0	0	0
MRV3-10p-A	33 (0.20)	28	1	1	1	0
MRV3-5p-B	30 (0.18)	24	3	2	1	0
MRV3-10p-B	33 (0.20)	28	6	0	0	0
F–RSV-MRV2	21 (0.12)	n/a	4	7	n/a	1
F–RSV-MRV2-5p-A	29 (0.17)	17	4	2	1	1
F–RSV-MRV2-10p-A	35 (0.20)	20	7	5	3	2
F–RSV-MRV2-5p-B	32 (0.18)	24	6	1	0	1
F–RSV-MRV2-10p-B	32 (0.18)	10	7	4	1	1

^a^ Sites not detected as variable in the preceding populations. ^b^ Relative to input plasmid sequence. ATU, additional transcription unit; not applicable, n/a.

**Table 3:** Differences between input plasmid sequences and consensus sequences of virus samples. For synonymous substitutions only the number of corresponding amino acid is shown. The sameness or equivalency (sameness in MuV backbone) of genomic positions is indicated with superscripts 1–9.

Virus Sample	Gene	Nucleotide Substitution *	Viral Variant %	Amino Acid Change *
MRV2 **	/	/	/	/
MRV2-5p	L	C9660T ^1^	74.66	Pro408Leu
MRV2-10p	HN	G8261A	78.66	Gly550Ser
	L	C9660T ^1^	100	Pro408Leu
	L	A13374C	79.43	Gly1646Pro
MRV3	L	C10572T ^1^	74.62	Pro408Leu
MRV3-5p-A	N	T1588C ^2^	93.80	n/a (481)
	N	T1599C ^3^	93.19	Leu485Ser
	N	T1601C	52.00	Tyr486His
	N	T1615C ^4^	91.20	n/a (490)
	HN	A8565T ^5^	56.17	Tyr347Phe
	L	C10572T ^1^	100	Pro408Leu
	L	T12047G ^6^	57.67	Phe900Val
MRV3-10p-A	L	C10572T^1^	100	Pro408Leu
MRV3-5p-B	N	T1588C ^2^	61.83	n/a (481)
	N	T1615C ^4^	55.99	n/a (490)
	L	C10572T ^1^	100	Pro408Leu
MRV3-10p-B	N	T1588C ^2^	100	n/a (481)
	N	T1599C ^3^	100	Leu485Ser
	N	T1615C ^4^	92.96	n/a (490)
	HN	A8565T ^5^	89.14	Tyr347Phe
	L	C10572T ^1^	100	Pro408Leu
	L	T12047G ^6^	89.33	Phe900Val
F–RSV-MRV2	N	T1599C ^3^	80.31	Leu485Ser
	N	T1615C ^4^	80.77	n/a (490)
	F–RSV	A3551G ^7^	79.02	Ile525Val
	L	C11604T ^1^	93.22	Pro408Leu
F–RSV-MRV2-5p-A	N	T1599C ^3^	74.38	Leu485Ser
	N	T1615C ^4^	69.85	n/a (490)
	F–RSV	A3551G ^7^	78.77	Ile525Val
	L	C11604T ^1^	94.41	Pro408Leu
F–RSV-MRV2-10p-A	N	T1599C ^3^	89.27	Leu485Ser
	N	T1615C ^4^	89.60	n/a (490)
	F–RSV	G2066T	87.86	Glu30stop
	F–RSV	A3551G ^7^	93.74	Ile525Val
	HN	A9152G	69.01	Asn199Asp
	L	C11604T ^1^	100	Pro408Leu
	L	G12584A	94.29	Asp735Asn
F–RSV-MRV2-5p-B	N	T1599C ^3^	88.12	Leu485Ser
	N	T1615C ^4^	100	n/a (490)
	N	A1902G ^8^	61.69	NCR
	F–RSV	A3551G ^7^	87.48	Ile525Val
	L	C11604T ^1^	94.66	Pro408Leu
	L	A13739G ^9^	63.71	Asn1120Asp
F–RSV-MRV2-10p-B	N	T1599C ^3^	98.28	Leu485Ser
	N	T1615C ^4^	98.14	n/a (490)
	N	A1902G ^8^	92.40	NCR
	F–RSV	A3551G ^7^	98.55	Ile525Val
	M	G5567A	67.79	n/a (120)
	L	C11604T ^1^	98.65	Pro408Leu
	L	A13739G ^9^	92.56	Asn1120Asp

* The first letter indicates a nucleotide or putative amino acid identity in the plasmid sequence; the second in the viral variant. ** No differences in consensus were detected. Not applicable, n/a; noncoding region, NCR.

## Data Availability

Data are contained within the article and its Supplementary Materials. Sequencing results were deposited in NCBI GenBank database (https://www.ncbi.nlm.nih.gov/genbank/), under acc. nos. MZ929423, MZ929424, MZ964861-MZ964864 and OK001340; and in the NCBI SRA database (https://www.ncbi.nlm.nih.gov/sra) BioProject ID PRJNA769261.

## Supplementary Materials

The following are available online at https://www.mdpi.com/article/10.3390/v13122550/s1, Figure S1. Schemes of plasmids’ segments corresponding to recombinant virus antigenomes; Figure S2. Mumps virus L protein models; Table S1. Enzymes and primers used in reverse transcription and PCR amplification; Table S2. Sequences of primers used in PCR amplifications; Table S3. Number of reads in NGS data sets (before and after quality control (QC), mapping and filtering) and mean coverage of genomic positions; Table S4. Positions and frequencies of nucleotide substitutions and corresponding amino acid changes in primary viral rescue stocks; only the analysis of mumps virus backbone region (not the inserts) is shown; Table S5. Positions and frequencies of heterogeneities in added genomic segments (not in mumps virus backbone) in primary rescue stocks of MRV3, F–RSV-MRV2, *mi*scrMRV3 and E1E2TMD-HCV-MRV2; Table S6. Variants detected during passaging of MRV2, MRV3 and F–RSV-MRV2.
