# Supplementary material for: Population Variability Generated during Rescue Process and Passaging of Recombinant Mumps Viruses
Source: Viruses. 2021 Dec 20;13(12):2550. doi: 10.3390/v13122550 (PMC8707793; doi:10.3390/v13122550)
Supplement: Supplementary file 1 [file viruses-13-02550-s001.zip › viruses-1455144-supplementary.pdf]

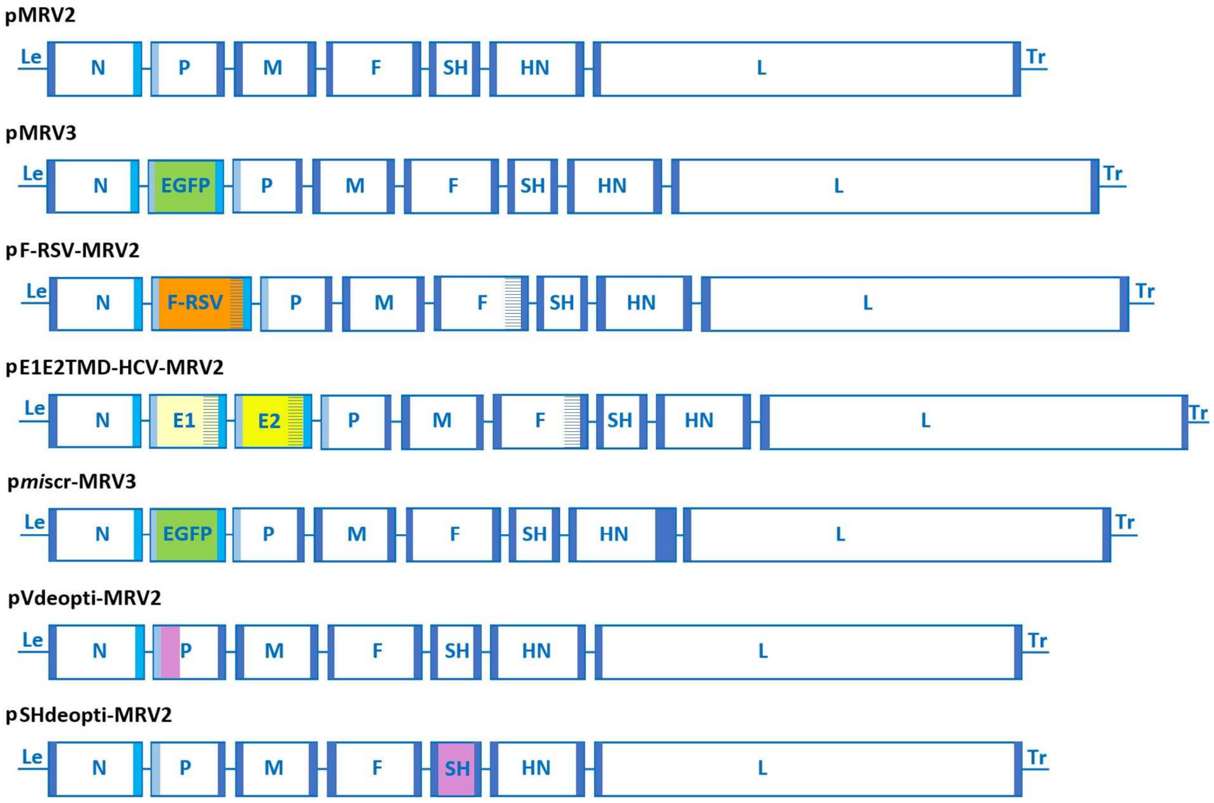

Figure S1. Plasmids' segments corresponding to recombinant virus antigenomes. The genomic regions in schemes are not drawn strictly to scale. Blue areas at the beginning and end of each gene indicate noncoding regions. P gene 5’ noncoding region (NCR) and N gene 3’ NCR are shown in different nuances in order to emphasize that they were included upstream and downstream of coding regions of additional transcription units (ATUs), respectively. In pF-RSV-MRV2 and pE1E2TMD-HCV-MRV2, striped areas indicate transmembrane and cytoplasmic region of mumps virus F glycoprotein, which was added to ectoplasmic domains of RSV F, and HCV E1 and E2 proteins. ATUs are shown in green, orange and yellow; regions with alternative synonymous sequences are shown in purple. The 3' NCR of HN gene in p*mi*sc-MRV3 is shown lengthened, to indicate addition of 84 nucleotides.

A) 408 Leu   B) 408 Pro

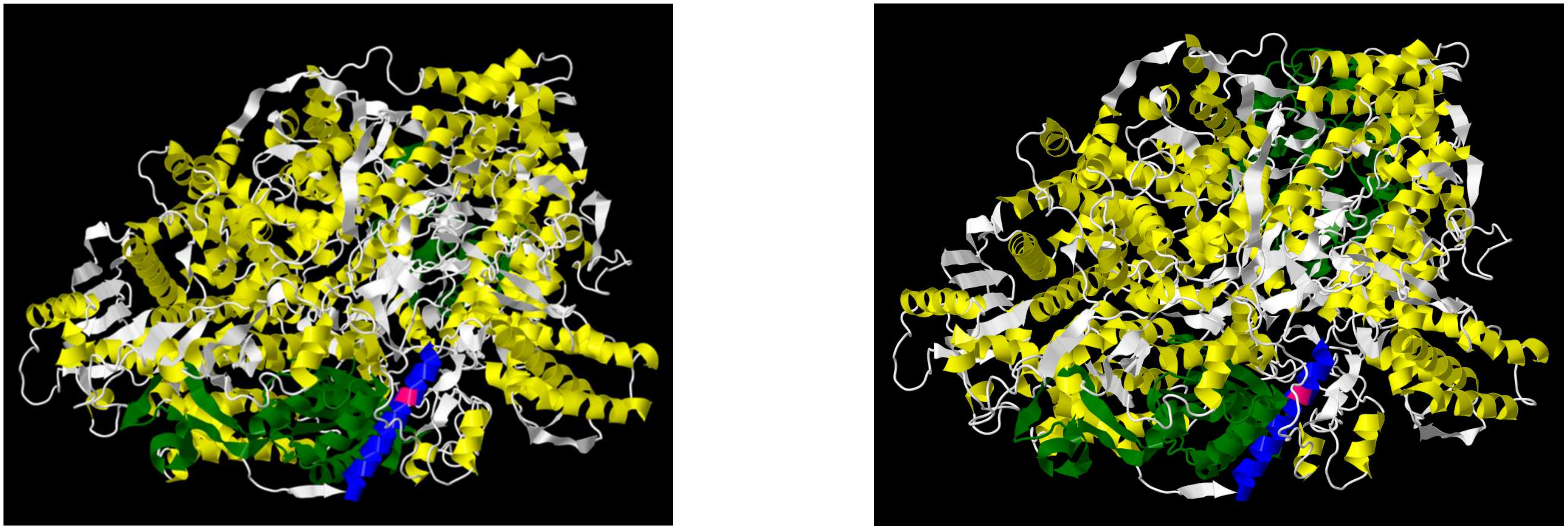

Figure S2. Mumps virus L protein models. The two L protein primary sequences differ only at amino acid 408 (leucine in figure A, proline in figure B). Models were generated using SWISS-MODEL homology-modelling server and visualized using Geneious Prime® 2019.2.3 software. Alpha-helix that extends from amino acid 394 to amino acid 416 is shown in blue, with amino acid 408 in red. Other α-helices are shown in yellow. Green colour indicates two L protein's catalytic regions: RNA-dependent RNA-polymerase and 2'-O-methyltransferase, shown in the lower left and upper right corner, respectively. Their positions were annotated according to UniProtKB entry P30929.

Table S1. Enzymes and primers used in reverse transcription and PCR amplification.

Sample	Reverse transcriptase	DNA polymerase	Primer pairs
**Plasmid sample**			
**pMRV2**	/	Phusion	N0_nextera/F2<br>M5/LZ6<br>LZ9/L12<br>MR9/L23<br>L24/L25_nextera
**Virus sample**			
**MRV2**	SuperScript III	Phusion	N0_nextera/F8<br>F15/LZ14<br>LZ13/L26<br>L9/L25_nextera
**MRV2-5p**	SuperScript III	Phusion	N0_nextera/F8<br>F15/LZ14<br>LZ13/L26<br>L9/L25_nextera
**MRV2-10p**	SuperScript III	Phusion	N0_nextera/F8<br>F15/LZ14<br>LZ13/L26<br>L9/L25_nextera
**MRV3**	M-MLV	Velocity	N0_nextera/M2<br>M1/LZ6<br>LZ9/L12<br>L13/L25_nextera
**MRV3-5p-Vero-A**	M-MLV	Velocity	N0_nextera/M2<br>M1/LZ6<br>LZ9/L12<br>L13/L25_nextera
**MRV3-10p-Vero-A**	M-MLV	Velocity	N0_nextera/M2<br>M1/LZ6<br>LZ9/L12<br>L13/L25_nextera
**MRV3-5p-Vero-B**	M-MLV	Velocity	N0_nextera/M2<br>M1/LZ6<br>LZ9/L12<br>L13/L25_nextera
**MRV3-10p-Vero-B**	M-MLV	Velocity	N0_nextera/M2<br>M1/LZ6<br>LZ9/L12<br>L13/L25_nextera
**F-RSV-MRV2**	M-MLV	Velocity	N0_nextera/M2<br>M1/LZ6<br>LZ9/L12<br>L13/L25_nextera
**F-RSV-MRV2-5p-A**	M-MLV	Velocity	N0_nextera/P6novi(-)<br>M1/LZ6<br>LZ9/L12<br>L13/L25_nextera
**F-RSV-MRV2-10p-A**	M-MLV	Velocity	N0_nextera/M2<br>M1/LZ6<br>LZ9/L12<br>L3InFusion/L25_nextera
**F-RSV-MRV2-5p-B**	M-MLV	Velocity	N0_nextera/P6novi(-)<br>M1/LZ6<br>LZ9/L12<br>L3InFusion/L25_nextera
**F-RSV-MRV2-10p-B**	M-MLV	Velocity	N0_nextera/P6novi(-)<br>M1/LZ6<br>LZ9/L12<br>L3InFusion/L25_nextera
**E1E2TMD-HCV-MRV2**	SuperScript III	Phusion	N0_nextera/P10rev<br>PstartHindIII/LZ6<br>LZ9/L12<br>MR9/L23<br>L24/L25_nextera
***mi* scrMRV3**	SuperScript III	Phusion	N0_nextera/M2<br>M1/LZ6<br>LZ9/L12<br>L13/L25_nextera<br>L3InFusion/L25_nextera
**Vdeopti-MRV2**	M-MLV	Velocity	N0_nextera/M2<br>M1/LZ6<br>LZ9/L12<br>L13/L25_nextera
**SHdeopti-MRV2**	M-MLV	Velocity	N0_nextera/M2<br>M1/LZ6<br>LZ9/L12<br>L13/L25_nextera

Table S2. Sequences of primers used in PCR amplifications.

Primer	Sequence
N0_nextera	TGCTGACTGCTCTGATCGTGGTAGCTGATAACCGTCAGTGTGATGTGATAGACCAAGGGGARAAAGAAGATGGGATATTG
LZ6	GCTCGCAATTTGTAACTAGG
L25_nextera	TGGTCATAATGCTGACTGCTGTGATCGTGGTAGTAGATTAGGGTCAGTGTGATGTACCAAGGGGAGAAAGTAAAATCAAT
M2	CGGAGCTGGCTTGAATCAGA
M1	GAATGCCAGACTCACGAGAA
LZ9	TTCTCTATCGGCCATCCACT
L12	GTCTCACCTCCAGTGAATACC
L13	GAAGAGGAACTCGCACAGTA
F8	CCAAGATCCTGTCTGGCAAT
L9	AAGGCTAATGCGAAGCACTC
P6novi(-)	GCCTTCTTGAGCCATGATT
L3InFusion	GAACTCGCACAGTATCTATTAGATCGTGAGGTGGTC
F15	GTATGACGCCTGCAGTGGTTC
LZ14	AGAGGAGTTCATACGGCCAC
LZ13	TTGTCTGTGCCTGGAATCAG
L26	ATTCAGAGCTGTATCAAGGTCA
P10rev	TTGTGGCCTTGCTTAACGAG
MR9	AGAAGGAGCAAGCTTATAAA
L23	CTGATGATTGGCCCTTTAGGA
L24	GGACTCCAAGCACAAGAA
F2	AGCTGGTTATCAAGGATCT
M5	TAGTCCATGCAGGCGGTCAC

Table S3. Number of reads in NGS datasets (before and after quality control (QC), mapping and filtering) and mean coverage of genomic positions.

	Sample	No. of reads	No. of reads after QC	% of mapped reads	No. of reads after removal of reads with more than 2 mismatches to reference	Mean coverage	Mean coverage st. dev.
**Plasmid (without PCR amplification)**							
	**pMRV2**	6,296,782	4,235,264	80.55	3,340,146	22,580	3,413
		1,254,252	828,654	80.77	653,179	4,390	681
		1,573,924	1,020,314	80.60	803,204	5,354	828
	**pMRV3**	1,007,806	846,414	81.96	690,167	4,932	640
	**pF-RSV-MRV2**	467,498	282,282	78.63	216,671	1,226	236
	**pE1E2TMD-HCV-MRV2**	735,198	592,176	82.47	485,533	3,150	378
	**p*mi* scr-MRV3**	564,174	467,978	82.58	384,411	2,712	364
	**pVdeopti-MRV2**	623,166	388,502	78.88	300,298	1,940	311
	**pSHdeopti-MRV2**	1,363,776	1,103,614	81.21	891,204	6,640	864
**Plasmid (PCR amplified)**							
	**pMRV2**	1,438,444	960,118	96.59	906,711	6,085	6,849
		1,025,268	678,290	96.68	640,980	4,285	4,778
		1,535,000	1,006,142	96.05	943,446	6,282	8,759
**Virus (PCR amplified)**							
	**MRV2**	482,232	389,916	98.48	381,766	2,818	1,239
	**MRV2-5p**	153,258	122,078	98.71	119,668	876	696
	**MRV2-10p**	470,624	380,568	98.82	373,647	2,763	928
	**MRV3**	921,748	582,382	80.22	459,807	2,863	2,256
	**MRV3-5p-A**	1,376,636	1,160,212	97.49	1,124,193	7,991	6,156
	**MRV3-10p-A**	874,732	580,050	95.43	540,871	3,422	3,308
	**MRV3-5p-B**	1,205,768	1,011,444	98.08	987,009	7,046	6,659
	**MRV3-10p-B**	1,385,982	1,169,316	98.50	1,146,054	8,222	7,491
	**F-RSV-MRV2**	1,084,550	931,740	96.18	892,550	6,119	3,768
	**F-RSV-MRV2-5p-A**	687,466	555,900	98.11	541,790	3,563	2,465
	**F-RSV-MRV2-10p-A**	933,032	772,470	96.84	743,227	4,962	3,028
	**F-RSV-MRV2-5p-B**	2,743,136	2,200,438	90.25	1,973,355	12,975	5,435
	**F-RSV-MRV2-10p-B**	959,368	795,954	95.33	754,686	5,070	1,462
	**E1E2TMD-HCV-MRV2**	832,430	670,134	97.83	650,854	4,192	3,678
	***mi* scr-MRV3**	540,448	430,592	97.89	418,633	2,875	1,463
	**Vdeopti-MRV2**	830,512	536,684	92.45	487,923	3,212	1,293
	**SHdeopti-MRV2**	411,670	320,652	97.30	311,208	2,259	1,542

Table S4. Positions and frequencies of nucleotide substitutions and corresponding amino acid changes in primary viral rescue stocks. The substitutions indicate the difference from input plasmid sequence (the first nucleotide/amino acid is the identity in plasmid). For synonymous substitutions only the number of corresponding amino acid is shown. ForMRV3, F-RSV-MRV2, E1E2TMD-HCV-MRV2 and *mi* scrMRV3 only the regions of mumps virus backbone (not the insert(s)) are included in the analysis, their genomic positions are adjusted to corresponding positions in MRV2. Substitutions present in viral population consensus sequence (present in more than 50% of reads) are indicated in bold.

	**MRV2**	**MRV3**	**F-RSV-MRV2**	**E1E2TMD-HCV-MRV2**	***mi* scrMRV3**	**Vdeopti-MRV2**	**SHdeopti-MRV2**
**total genome length (including the insert(s))**	**15384**	**16296**	**17328**	**17562**	**16380**	**15384**	**15384**
**no. of variable sites in MuV backbone**	**4**	**18**	**14**	**9**	**6**	**9**	**13**
**Gemomic region (length)**							
**leader (55)**							
**N gene (1851)**							
5' noncoding region (90)		G131A (1.7%)					
coding region (1650)	G1683A; Arg513Gln (1.6%)	T1588C; 481 (5.5%)<br>T1615C; 490 (2.3%)	T436G; 97 (1.0%)<br>T647C; Tyr168His (1.8%)<br>**T1599C; Leu485Ser (80.3%)**<br>**T1615C; 490 (80.8%)**				
3' noncoding region (111)			C1834T (2.8%)<br>A1902G (32.2%)		C1834T (1.7%)		
**intergenic region (2)**							
**P gene (1318)**							
5' noncoding region (70)			T1961G (3.3%)				
P coding region (1174)				C2627T; Leu97Phe (1.0%)		A2139C; Gln54Pro (1.6%)	G2085A; Ser36Asn (8.2%)
V coding region (675)				C2627T; Leu97Phe (1.0%)		A2139C; Gln54Pro (1.6%)	G2085A; Ser36Asn (8.2%)
I coding region (512)				C2627T; Leu97Phe (1.0%)		A2139C; Gln54Pro (1.6%)	G2085A; Ser36Asn (8.2%)
3' noncoding region (74)					T3220A (1.0%)		
**intergenic region (1)**							
**M gene (1254)**							
5' noncoding region (36)							
coding region (1128)		C3902A; Cys213stop (1.1%)<br>A4282G; Lys340Arg (2.8%)	G4051A; Arg263His (4.8%)			A3396T; Ile45Leu (1.0%)<br>C3902A; Cys213stop (1.0%)	
3' noncoding region (90)							
**F gene (1729)**							
5' noncoding region (64)							
coding region (1617)	T4681G; Leu25Val (2.4%)			C5814T; 423 (11.0%)		A5068G; Ile175Val (16.4%)	
3' noncoding region (48)							
**intergenic region (7)**							
**SH gene (316)**							
5' noncoding region (50)							
coding region (174)	A6354G; Ile29Met (1.1%)						
3' noncoding region (92)						G6527A (1.0%)	G6527A (1.6%)
**intergenic region (2)**							
**HN gene (1893)**							
5' noncoding region (78)							
coding region (1749)		T7593C; Leu327Ser (1.1%)<br>G7692A; Arg360His (1.9%)<br>A8154G; Asn514Ser (2.2%)	A6829C; 72 (8.1%)	T6870C; Val86Ala (1.7%)		T8222C; Phe537Leu (3.7%)	
3' noncoding region (66)		G8422A (1.1%)		T8419C (2.5%)			
**intergenic region (1)**							
**L gene (6931)**							
5' noncoding region (8)							
coding region (6786)	C9660T; Pro408Leu (35.7%)	A8569G; 44 (3.7%)<br>A8920T; 161 (1.8%)<br>**C9660T; Pro408Leu (74.6%)**<br>A10589C; Asn718His (1.8%)<br>T10646C; Phe737Leu (3.5%)<br>C11538T; Pro1034Leu (1.7%)<br>T11780A; Leu1115Met (1.6%)<br>G12758A; Glu1441Lys (2.3%)<br>G14191A; 1918 (3.6%)	G8603T; Glu56stop (38.6%)<br>T8815A; Asp126Glu (3.8%)<br>C9659T; Pro408Ser (6.6%)<br>**C9660T; Pro408Leu (93.2%)**<br>C13924A; 1829 (1.1%)	**C8447C; Leu4Ile (89.3%)**<br>G8959T; Glu174Asp (2.0%)<br>C9566T; Gln377stop (4.1%)<br>**C9660T; Pro408Leu (100%)**<br>T11274A; Leu946Gln (4.0%)	C8678T; Arg81Cys (1.3%)<br>C9660T; Pro408Leu (37.7%)<br>T11379C; Ile981Thr (1.3%)<br>G12758A; Glu1441Lys (1.2%)	C9660T; Pro408Leu (9.8%)<br>G12758A; Glu1441Lys (2.6%)<br>A13251G; Gln1605Arg (1.1%)	T8624G; Leu63Val (3.2%)<br>A8786G; Thr117Ala (1.2%)<br>**C9660T; Pro408Leu (85.8%)**<br>G9905A; Gly490Arg (2.5%)<br>C9998A;Leu521Ile (4.2%)<br>T11235G;Ile933Ser (1.9%)<br>G11782T; Leu1115Phe (1.4%)<br>T11797A; Asn1120Lys (1.2%)<br>T11936A; Leu1167Ile (2.0%)<br>T14354A; Phe1973Ile (3.2%)<br>T14562G; Ile2042Ser (3.3%)
3' noncoding region (137)							
**trailer (24)**							

Table S5. Positions and frequencies of variable sites in added genomic segments (ATUs) in primary rescue stocks of MRV3, F-RSV-MRV2, *mi* scrMRV3 and E1E2TMD-HCV-MRV2. The nucleotide and amino acid changes are indicated. For synonymous substitutions only the number of corresponding amino acid is shown. Substitution present in the viral population consensus sequence (present in more than 50% of reads) is indicated in bold.

	**MRV3**	**F-RSV-MRV2**	**E1E2TMD-HCV-MRV2**	***mi* scrMRV3**
**insert length**	**912**	**1944**	**2178**	**996**
**no. of variable sites**	**2**	**7**	**1**	**0**
**region (length)**				
**insert 1**				
**ATU1**				
5' noncoding region (70)	T1952C (1.4%)	none	none	none
coding region<br>(720 for MRV3 and *mi* scrMRV3,<br>1758 for F-RSV-MRV2,<br>645 for E1E2TMD-HCV-MRV2)	G2507A; Val177Met (1.1%)	A2251G; 91 (1.1%)<br>A2673G; Glu232Gly (1.0%)<br>T2720C; Ser248Pro (1.3%)<br>C3016T; 346 (2.5%)<br>**A3551G; Ile525Val (79.0%)**<br>G3581A; Val535Met (1.0%)	none	none
3' noncoding region<br>(120 for MRV3 and *mi* scrMRV3,<br>114 for F-RSV-MRV2,<br>111 for E1E2TMD-HCV-MRV2)	none	A3846G (1.2%)	none	none
**intergenic region ATU1-ATU2 (2)**	n/a	n/a	none	n/a
**ATU2**				
5' noncoding region (70)	n/a	n/a	T2751C (4.1%)	n/a
coding region (1167)	n/a	n/a	none	n/a
3' noncoding region (111)	n/a	n/a	none	n/a
**intergenic region insert 1-P gene (2)**	none	none	none	none
**insert 2**				
3' HN noncoding region (84)	n/a	n/a	n/a	none

n/a, not applicable

Table S6. Variants detected during passaging of MRV2, MRV3 and F-RSV-MRV2. As references, input plasmid sequences were used.

viral sample	genomic position	genomic region	viral variant	plasmid	viral variant percentage
**MRV2**	1683	N gene; CR	A	G	1.625
	4618	F gene; CR	G	T	2.434
	6354	SH gene; CR	G	A	1.125
	9660	L gene; CR	T	C	35.66
**MRV2-5p**	577	N gene; CR	G	A	1.296
	860	N gene; CR	G	T	1.733
	1793	N gene; CR	C	T	6.583
	2728	P gene; CR	G	A	3.135
	5024	F gene; CR	G	A	1.258
	6354	SH gene; CR	G	A	1.230
	6527	SH gene; 3' NCR	A	G	1.603
	8261	HN gene; CR	A	G	5.417
	9660	L gene; CR	T	C	74.66
	11274	L gene; CR	A	T	1.149
	11657	L gene; CR	G	A	1.022
	13374	L gene; CR	C	A	4.612
	14324	L gene; CR	C	T	1.156
**MRV2-10p**	1793	N gene; CR	C	T	26.76
	2349	P gene; CR	T	G	1.457
	2728	P gene; CR	G	A	1.267
	3220	P gene; 3' NCR	A	T	1.008
	7101	HN gene; CR	C	T	1.257
	8261	HN gene; CR	A	G	78.66
	9660	L gene; CR	T	C	100
	12254	L gene; CR	T	G	1.002
	13374	L gene; CR	C	A	79.43
	14864	L gene; CR	G	A	1.047
**MRV3**	131	N gene; 5' NCR	A	G	1.724
	1588	N gene; CR	C	T	5.466
	1615	N gene; CR	C	T	2.314
	1952	EGFP gene; 5' NCR	C	T	1.358
	2507	EGFP gene; CR	A	G	1.138
	4814	M gene; CR	A	C	1.122
	5194	M gene; CR	G	A	2.757
	8505	HN gene; CR	C	T	1.137
	8604	HN gene; CR	A	G	1.922
	9066	HN gene; CR	G	A	2.203
	9334	HN gene; 3' NCR	A	G	1.062
	9481	L gene; CR	G	A	3.726
	9832	L gene; CR	T	A	1.829
	10572	L gene; CR	T	C	74.62
	11501	L gene; CR	C	A	1.845
	11558	L gene; CR	C	T	3.,483
	12450	L gene; CR	T	C	1.744
	12692	L gene; CR	A	T	1.567
	13670	L gene; CR	A	G	2.251
	15103	L gene; CR	A	G	3.562
**MRV3-5p-A**	135	N gene; 5' NCR	T	C	2.649
	1588	N gene; CR	C	T	93.8
	1599	N gene; CR	C	T	93.19
	1601	N gene; CR	C	T	52.000

	1615	N gene; CR	C	T	91.2
	3274	P gene; CR	C	T	17.89
	3305	P gene; CR	C	T	17.75
	3494	P gene; CR	C	T	19.110
	3587	P gene; CR	C	A	14.500
	3598	P gene; CR	G	A	1.856
	3735	P gene; CR	A	G	1.540
	3750	P gene; CR	A	G	2.083
	5424	F gene; 5' NCR	G	T	3.577
	6514	F gene; CR	C	A	5.024
	8144	HN gene; CR	C	T	17.94
	8181	HN gene; CR	G	A	6.163
	8196	HN gene; CR	G	A	1.601
	8226	HN gene; CR	T	A	2.832
	8506	HN gene; CR	A	G	3.898
	8561	HN gene; CR	T	C	1.572
	8565	HN gene; CR	T	A	56.17
	9220	HN gene; CR	G	T	1.201
	10572	L gene; CR	T	C	100
	11475	L gene; CR	A	G	1.020
	11525	L gene; CR	T	C	3.554
	11546	L gene; CR	C	A	2.087
	12047	L gene; CR	G	T	57.67
	13551	L gene; CR	C	T	1.246
	14177	L gene; CR	A	G	2.392
	15658	L gene; CR	G	A	1.479
**MRV3-10p-A**	409	N gene; CR	C	T	17.68
	1588	N gene; CR	C	T	44.29
	1599	N gene; CR	C	T	21.88
	1615	N gene; CR	C	T	43.98
	2476	EGFP gene; CR	T	C	17.300
	3090	P gene; CR	T	C	1.249
	3116	P gene; CR	A	G	1.221
	3748	P gene; CR	G	A	31.78
	4252	M gene; CR	C	T	14.26
	4305	M gene; CR	G	A	1.016
	4497	M gene; CR	T	G	1.871
	7422	SH gene; 3' NCR	T	C	2.116
	7484	HN gene; 5' NCR	G	A	1.107
	7568	HN gene; CR	C	T	3.757
	7810	HN gene; CR	G	A	1.202
	7949	HN gene; CR	C	A	1.420
	8181	HN gene; CR	G	A	4.386
	8196	HN gene; CR	C	A	19.43
	8232	HN gene; CR	A	G	20.46
	8570	HN gene; CR	T	C	2.245
	9089	HN gene; CR	A	C	20.39
	9467	L gene; CR	A	G	16.85
	10080	L gene; CR	T	C	13.25
	10309	L gene; CR	G	A	22.070
	10572	L gene; CR	T	C	100
	11425	L gene; CR	T	G	22.040
	12477	L gene; CR	T	A	1.928
	12924	L gene; CR	T	G	16.19
	13449	L gene; CR	T	A	1.027

	14480	L gene; CR	G	A	15.81
	15915	L gene; CR	T	G	8.645
	16182	L gene; 3' NCR	T	C	1.563
	16183	L gene; 3' NCR	G	A	1.659
**MRV3-5p-B**	409	N gene; CR	C	T	1.203
	1401	N gene; CR	A	C	1.132
	1588	N gene; CR	C	T	61.83
	1599	N gene; CR	C	T	43.24
	1601	N gene; CR	C	T	4.688
	1615	N gene; CR	C	T	55.99
	1952	EGFP gene; 5' NCR	C	T	2.514
	2476	EGFP gene; CR	T	C	2.199
	3748	P gene; CR	G	A	23.24
	4814	M gene; CR	A	C	1.171
	7422	SH gene; 3' NCR	T	C	2.039
	7439	SH gene; 3' NCR	A	G	1.257
	7805	HN gene; CR	T	C	1.788
	7985	HN gene; CR	C	A	1.463
	8196	HN gene; CR	C	A	2.124
	8232	HN gene; CR	A	G	6.290
	9224	HN gene; CR	A	G	1.458
	9467	L gene; CR	A	G	5.759
	9840	L gene; CR	T	G	1.319
	10080	L gene; CR	T	C	16.800
	10309	L gene; CR	G	A	2.820
	10572	L gene; CR	T	C	100
	10809	L gene; CR	G	A	2.799
	11425	L gene; CR	T	G	33.61
	11558	L gene; CR	C	T	1.914
	12261	L gene; CR	C	A	1.354
	12924	L gene; CR	T	G	5.548
	13449	L gene; CR	T	A	1.834
	14480	L gene; CR	G	A	6.567
	15915	L gene; CR	T	G	14.54
**MRV3-10p-B**	135	N gene; 5' NCR	T	C	2.564
	1588	N gene; CR	C	T	100
	1599	N gene; CR	C	T	100
	1615	N gene; CR	C	T	92.96
	3274	P gene; CR	C	T	1.580
	3305	P gene; CR	C	T	1.453
	3312	P gene; CR	G	A	1.035
	3735	P gene; CR	A	G	2.322
	3750	P gene; CR	A	G	2.091
	4814	M gene; CR	A	C	1.181
	5424	F gene; 5' NCR	G	T	5.667
	6326	F gene; CR	G	A	1.059
	6514	F gene; CR	C	A	1.904
	8144	HN gene; CR	C	T	1.460
	8181	HN gene; CR	G	A	2.204
	8181	HN gene; CR	T	A	1.440
	8196	HN gene; CR	G	A	1.425
	8226	HN gene; CR	T	A	2.196
	8324	HN gene; CR	A	G	3.725
	8506	HN gene; CR	A	G	2.240
	8516	HN gene; CR	G	A	8.016

	8565	HN gene; CR	T	A	89.14
	9182	HN gene; CR	G	A	23.33
	9184	HN gene; CR	G	T	3.184
	9220	HN gene; CR	G	T	1.583
	10572	L gene; CR	T	C	100
	11525	L gene; CR	T	C	2.002
	12047	L gene; CR	G	T	89.33
	13204	L gene; CR	G	A	4.458
	13670	L gene; CR	A	G	1.022
	14177	L gene; CR	A	G	1.141
	14767	L gene; CR	C	T	3.391
	15658	L gene; CR	G	A	1.032
**F-RSV-MRV2**	436	N gene; CR	G	T	1.042
	647	N gene; CR	C	T	1.772
	1599	N gene; CR	C	T	80.31
	1615	N gene; CR	C	T	80.77
	1834	N gene; 3' NCR	T	C	28.149
	1902	N gene; 3' NCR	G	A	32.200
	2251	F-RSV gene; CR	G	A	1.066
	2673	F-RSV gene; CR	G	A	1.044
	2720	F-RSV gene; CR	C	T	1.335
	3016	F-RSV gene; CR	T	C	2.500
	3551	F-RSV gene; CR	G	A	79.02
	3581	F-RSV gene; CR	A	G	1.023
	3846	F-RSV gene; 3' NCR	G	A	1.174
	3905	P gene; 5' NCR	G	T	3.336
	5995	M gene; CR	A	G	4.780
	8773	HN gene; CR	C	A	8.062
	10547	L gene; CR	T	G	38.61
	10759	L gene; CR	A	T	3.774
	11603	L gene; CR	T	C	6.609
	11604	L gene; CR	T	C	93.22
	15868	L gene; CR	A	C	1.095
**F-RSV-MRV2-5p-A**	436	N gene; CR	G	T	5.978
	1590	N gene; CR	C	T	13.55
	1599	N gene; CR	C	T	74.38
	1615	N gene; CR	C	T	69.85
	2066	F-RSV gene; CR	T	G	41.99
	3551	F-RSV gene; CR	G	A	78.77
	3905	P gene; 5' NCR	G	T	5.442
	4062	P gene; CR	C	T	3.409
	5008	P gene; CR	G	A	8.969
	5846	M gene; CR	A	C	1.127
	5995	M gene; CR	A	G	18.54
	6391	M gene; 3' NCR	A	G	4.647
	7273	F gene; CR	A	G	1.185
	7855	F gene; CR	A	G	1.281
	8773	HN gene; CR	C	A	3.360
	8795	HN gene; CR	G	A	3.790
	9152	HN gene; CR	G	A	1.007
	10206	HN gene; CR	A	G	1.993
	10547	L gene; CR	T	G	18.100
	10759	L gene; CR	A	T	17.76
	11153	L gene; CR	G	A	1.023
	11603	L gene; CR	T	C	5.595

	11604	L gene; CR	T	C	94.41
	11947	L gene; CR	T	G	1.950
	12161	L gene; CR	A	G	1.069
	12475	L gene; CR	A	G	1.461
	12584	L gene; CR	A	G	40.79
	13218	L gene; CR	A	T	2.311
	16379	L gene; CR	A	G	4.608
**F-RSV-MRV2-10p-A**	46	leader	G	A	25.38
	870	N gene; CR	A	G	2.153
	1590	N gene; CR	C	T	2.195
	1599	N gene; CR	C	T	89.27
	1615	N gene; CR	C	T	89.6
	1655	N gene; CR	C	G	1.088
	1793	N gene; CR	C	T	3.033
	1834	N gene; 3' NCR	T	C	1.774
	1902	N gene; 3' NCR	G	A	3.822
	1979	F-RSV gene; CR	G	A	1.407
	2066	F-RSV gene; CR	T	G	87.86
	2225	F-RSV gene; CR	T	C	1.225
	3264	F-RSV gene; CR	A	G	1.821
	3551	F-RSV gene; CR	G	A	93.74
	3874	P gene; 5' NCR	C	T	2.483
	4339	P gene; CR	C	T	1.100
	4342	P gene; CR	A	T	1.041
	4360	P gene; CR	G	A	1.356
	4750	P gene; CR	G	A	1.366
	5846	M gene; CR	A	C	1.129
	5995	M gene; CR	A	G	2.534
	6140	M gene; CR	A	G	1.018
	8773	HN gene; CR	C	A	1.385
	8795	HN gene; CR	G	A	4.153
	9152	HN gene; CR	G	A	69.01
	10206	HN gene; CR	A	G	1.593
	10759	L gene; CR	A	T	1.763
	10777	L gene; CR	T	A	4.098
	11604	L gene; CR	T	C	100
	12475	L gene; CR	A	G	1.248
	12546	L gene; CR	G	A	1.196
	12584	L gene; CR	A	G	94.29
	13340	L gene; CR	C	A	1.744
	13693	L gene; CR	A	G	5.570
	14869	L gene; CR	T	C	4.497
**F-RSV-MRV2-5p-B**	647	N gene; CR	C	T	1.013
	1599	N gene; CR	C	T	88.12
	1615	N gene; CR	C	T	100
	1902	N gene; 3' NCR	G	A	61.69
	3551	F-RSV gene; CR	G	A	87.48
	3905	P gene; 5' NCR	G	T	6.112
	4040	P gene; CR	C	A	1.251
	4798	P gene; CR	C	T	1.083
	5445	M gene; CR	A	C	4.015
	5567	M gene; CR	A	G	23.060
	5846	M gene; CR	A	C	1.707
	5995	M gene; CR	A	G	2.044
	7093	F gene; CR	A	G	1.004

	8773	HN gene; CR	C	A	3.319
	9052	HN gene; CR	A	G	1.127
	9228	HN gene; CR	G	A	1.146
	9749	HN gene; CR	G	T	2.521
	10051	HN gene; CR	G	T	1.073
	10106	HN gene; CR	T	C	2.060
	10330	HN gene; 3' NCR	T	C	1.828
	10547	L gene; CR	T	G	7.247
	10759	L gene; CR	A	T	1.872
	11229	L gene; CR	A	T	1.275
	11603	L gene; CR	T	C	5.259
	11604	L gene; CR	T	C	94.66
	11757	L gene; CR	T	G	1.187
	11960	L gene; CR	T	G	2.972
	12858	L gene; CR	C	A	2.921
	13218	L gene; CR	A	T	1.137
	13739	L gene; CR	G	A	63.71
	13845	L gene; CR	T	G	1.645
	15360	L gene; CR	T	A	1.304
**F-RSV-MRV2-10p-B**	82	N gene; 5' NCR	C	T	1.187
	647	N gene; CR	C	T	3.438
	1599	N gene; CR	C	T	98.28
	1615	N gene; CR	C	T	98.14
	1902	N gene; 3' NCR	G	A	92.4
	1905	N gene; 3' NCR	G	A	3.083
	3016	F-RSV gene; CR	T	C	3.229
	3506	F-RSV gene; CR	C	G	1.189
	3551	F-RSV gene; CR	G	A	98.55
	3846	F-RSV gene; 3' NCR	G	A	1.061
	3905	P gene; 5' NCR	G	T	1.147
	4040	P gene; CR	C	A	5.196
	5445	M gene; CR	A	C	1.326
	5446	M gene; CR	G	A	3.001
	5567	M gene; CR	A	G	67.79
	5846	M gene; CR	A	C	1.085
	6529	F gene; CR	C	T	2.473
	7093	F gene; CR	A	G	39.37
	9052	HN gene; CR	A	G	1.430
	9177	HN gene; CR	T	C	3.711
	9228	HN gene; CR	G	A	38.44
	9749	HN gene; CR	G	T	1.454
	10051	HN gene; CR	G	T	3.643
	10106	HN gene; CR	T	C	3.730
	10205	HN gene; CR	A	G	1.077
	10330	HN gene; 3' NCR	T	C	1.109
	11603	L gene; CR	T	C	1.301
	11604	L gene; CR	T	C	98.65
	11960	L gene; CR	T	G	1.020
	12858	L gene; CR	C	A	46.98
	13739	L gene; CR	G	A	92.56
	16255	L gene; CR	G	T	4.980

CR, coding region
NCR, noncoding region
